# circ-0001454 alleviates asthma airway inflammation and remodeling via sponging miR-770-5p and regulating cbl-b

**DOI:** 10.3389/fcell.2025.1566223

**Published:** 2025-04-08

**Authors:** Ruobai Liu, Yilan Song, Zhiguang Wang, Longzhu Dai, Qiaoyun Bai, Yan Li, Hongmei Piao, Chongyang Wang, Guanghai Yan

**Affiliations:** ^1^ Jilin Key Laboratory for Immune and Targeting Research on Common Allergic Diseases, Yanbian University, Yanji, China; ^2^ Department of Anatomy, Histology and Embryology, Yanbian University Medical College, Yanji, China; ^3^ Department of Respiratory Medicine, Affiliated Hospital of Yanbian University, Yanji, China

**Keywords:** asthma, airway inflammation, airway remodeling, circ-0001454, miR-770-5p, Cbl-b

## Abstract

Bronchial asthma is a chronic inflammatory disease that has long been a severe threat to human physical and mental health. Circular RNAs (circRNAs) and microRNAs (miRNAs) are involved in regulating various processes in asthma. However, the mechanisms by which these molecules influence the pathophysiological processes of asthma through target gene regulation remain unclear. Our study found that inhibition of miR-770-5p alleviated airway inflammation and remodeling in asthmatic mice. Furthermore, bioinformatics analysis revealed that circ-0001454 harbors binding sites for miR-770-5p, acting as a sponge to adsorb miR-770-5p and function as a competing endogenous RNA (ceRNA), thereby negatively regulating the expression of miR-770-5p. Circ-0001454 not only alleviated airway inflammation and remodeling in asthmatic mice, but also participated in modulating the HDM-induced inflammatory response in BEAS-2B cells. It mitigated bronchial epithelial cell inflammatory damage, reduced oxidative stress, apoptosis, and mitochondrial membrane potential loss. Mechanistically, we observed that circ-0001454 partially alleviated the inflammatory damage of epithelial cells caused by miR-770-5p overexpression excessive reactive oxygen species (ROS) production, apoptosis, and mitochondrial membrane potential disruption. Lastly, we found that circ-0001454 targets miR-770-5p and participates in regulating the expression of cbl-b, which in turn modulates the levels of EGFR, AKT1, and MAPK1 proteins, thereby alleviating inflammation in airway epithelial cells. These findings reveal the role of miR-770-5p in asthma, and how circ-0001454, by binding to miR-770-5p and targeting the gene cbl-b, contributes to the attenuation of airway inflammation, reduction of ROS levels, inhibition of apoptosis, and restoration of mitochondrial membrane potential. This regulation of cbl-b, EGFR, AKT1, and MAPK1 suggests new potential therapeutic targets for asthma treatment.

## Introduction

Asthma, a prevalent chronic inflammatory disorder, is characterized by the collective involvement of inflammatory cells, airway structural components, and cellular elements. This condition is predominantly marked by persistent inflammation within the airways, evidenced by substantial infiltration of inflammatory cells into lung tissues and the augmented secretion of inflammatory mediators. These inflammatory processes are central to the emergence of airway hyperresponsiveness, prolonged airway sensitivity, and structural modifications, which are collectively recognized as airway remodeling, a defining feature of asthma (Cevhertas et al., 2020). Chronic airway inflammation’s development is intricately linked to oxidative stress, significantly influenced by environmental pollutants and allergens. These factors sustain the activation of airway inflammatory and epithelial cells, leading to an overproduction of ROS. The excessive ROS levels prompt the activation of intracellular pro-inflammatory signaling pathways, thereby intensifying chronic airway inflammation (Liang et al., 2020; Huang et al., 2019). Additionally, high ROS levels are implicated in mitochondrial dysfunction, which contributes to the release of a variety of inflammatory cytokines and amplifies oxidative stress damage. Particularly, mitochondrial ROS has been shown to activate calcium/calmodulin-dependent kinase II (CaMKII) and the inflammasome component NOD-like receptor thermal protein domain-associated protein 3 (NLRP3). These activations play crucial roles in mediating the expression of genes associated with inflammation, thereby escalating the severity of inflammatory damage to airway cells (Huang et al., 2023). Mitochondrial anomalies, characterized by oxidative stress, inflammation, and apoptosis, are significant contributors to the pathogenesis of asthma. Addressing mitochondrial dysfunction and the subsequent inhibition of oxidative stress processes are therefore vital for mitigating the inflammatory responses in asthmatic airways (Michaeloudes et al., 2022; Cai et al., 2022).

Recent research into microRNAs (miRNAs) has increasingly highlighted their role in regulating various processes within asthma pathophysiology. These miRNAs engage with numerous target genes, influencing crucial biological functions including cell growth, tissue differentiation, cellular proliferation, embryonic development, and apoptosis (Chen et al., 2019). In particular, studies have demonstrated that suppression of miR-21 mitigates airway inflammation in ovalbumin-treated mice. This effect appears to stem from the downregulation of inflammatory mediators such as IL-4, IL-5, and IL-13, achieved through the inhibition of the miR-21-TGF-β1-Smad7 signaling pathway, thus reducing both inflammation and remodeling in the airways (Hur et al., 2021). Additionally, Noyan S et al. have shown that overexpression of miR-770-5p can restore E-Cadherin expression in MDA-MB-231 cells by directly targeting DNMT3A (Noyan et al., 2021). Similarly, Wang et al. demonstrated that the knockdown of miR-770-5p reduces podocyte apoptosis and inflammatory response by targeting TIMP3, suggesting miR-770-5p as a potential therapeutic target for diabetic nephropathy (Wang and Li, 2020). However, the specific influence of miR-770-5p on the inflammatory response in asthma remains to be elucidated.

Extensive research underscores the diverse roles of miRNAs, not only in influencing cell proliferation, migration, inflammation, and oxidative stress but also in their function within circRNAs. These circRNAs play critical roles in immune and allergic diseases, participating in the regulation of diseases such as inflammatory bowel disease (Gong and Maquat, 2011), osteoarthritis (Hadjicharalambous and Lindsay, 2019), and asthma (Zhang et al., 2018). The discovery of the first circular RNA (circRNA) in 1976 through electron microscopy marked a significant milestone. CircRNAs are characterized by their covalently closed loop structure, formed by the joining of the 3′ and 5′ ends (Sanger et al., 1976). This unique configuration grants them a longer half-life and greater resistance to degradation by ribonuclease R (RNase R), enabling stable presence in the cytoplasm of eukaryotic cells (Harland and Misher, 1988). With advances in high-throughput sequencing technologies, it has been revealed that circRNAs contribute to the epigenetic regulation of asthma, impacting the disease’s pathological processes. Functionally, circRNAs are enriched with microRNA (miRNA) binding sites, serving as “sponges” to sequester miRNAs, thereby shielding target genes from miRNA-induced suppression and modulating gene expression (Lin et al., 2021; Liu et al., 2019; Chen et al., 2022). For instance, circRNA406961 has been found to modulate the bronchial epithelial inflammatory response to PM2.5 exposure. It achieves this by interacting with interleukin enhancer-binding factors and downregulating IL-6 and IL-8 via activation of the STAT3/JNK pathway, thus exerting an anti-inflammatory effect (Jia et al., 2020). Zeng et al. performed RNA sequencing on cigarette smoke extract-treated airway epithelial cells and constructed a circRNA-miRNA-mRNA regulatory network to explore the underlying mechanisms. They found that differentially expressed circRNAs modulate the expression of genes involved in multiple signaling pathways. Their study suggests that smoking may alter the expression profiles of circRNAs and mRNAs in airway epithelial cells, thereby contributing to the pathogenesis of chronic obstructive pulmonary disease (COPD) (Vo et al., 2019).

Further studies, such as those by Huang et al., have illustrated that circ-ERBB2 can impact the proliferation and migration of airway smooth muscle cells by competitively binding to miR-98-5p (Huang et al., 2021). The cbl-b protein, integral to the cbl family, is characterized by its RING-finger domain and operates as an E3 ubiquitin ligase. This enzyme is essential for ubiquitination and subsequent degradation of specified target proteins, facilitating the negative modulation of cellular signaling. In addition to direct interactions, the cbl-b acts as a pivotal connector, bridging proteins to modulate signaling pathways indirectly (Liyasova et al., 2015). Its role has been extensively studied in various pulmonary disorders, encompassing lung tumors, acute lung injuries, pulmonary infections, and the regulation of lung inflammation. Research by Qiao et al. (2014) highlighted that in a murine model of allergic asthma, the absence of cbl-b leads to pronounced airway inflammation. This inflammation is notably driven by dysregulated immune responses, specifically Th2 and Th9 types, during the process of OVA immunization. The study further detailed that in OVA-induced asthma models, the deletion of cbl-b gene enhances the recruitment of neutrophils and eosinophils into the lung’s airways. Additionally, these knockout mice displayed increased mucus production within the lungs and elevated serum levels of inflammatory and immune-modulating cytokines, including IFN-γ, IL-10, IL-13, and eotaxin (Oh et al., 2011). Such findings underscore the critical regulatory role of cbl-b in immune response dynamics within the respiratory system, particularly in the context of allergic asthma.

In this study, we extensively investigated the potential of circ-0001454 as a modulator in asthma regulation through a comprehensive experimental approach. By examining its interactions with miR-770-5p and cbl-b, this research aimed to illuminate the role of circular RNA in asthma and suggest novel preventative and therapeutic targets.

## Materials and methods

### Animals, grouping, establishment, and treatment of animal models

In this investigation, SPF BALB/c male mice, aged 5–6 weeks and weighing around 20 ± 5 g, were procured from Yanbian University. A total of 40 mice were selected to ensure robust statistical analysis. These mice were maintained in a controlled laboratory environment where the temperature was regulated at 20°C ± 2°C and relative humidity was kept between 50% and 60%. All animals had consistent access to a sufficient supply of clean food and water throughout the duration of the study. The experimental procedures were strictly followed in compliance with the guidelines approved by the Medical Ethics Committee of Yanbian University, under approval number YD20240025, ensuring ethical standards were met for animal welfare.

After 1 week of adaptive feeding, mice in the circ-0001454 experimental group were randomly divided into four groups: control, HDM, LV-NC, and LV-circ-0001454 treatment groups (Ribobio, Guangzhou, China), with 5 mice per group, totaling 20 mice. Similarly, mice in the miR-770-5p experimental group were randomly assigned to four groups: control, HDM, miR-770-5p-NC, and miR-770-5p-antagomir groups (Ribobio, Guangzhou, China), with 5 mice per group, totaling 20 mice. Except for the control group, all other groups were sensitized by intratracheal instillation of 50 μL HDM working solution (XPB46D3A4, Greer Laboratories, USA) on days 1, 7, and 14, while the control group received an equal volume of PBS. Starting from day 21, mice were challenged by intratracheal instillation of 50 μL HDM working solution for three consecutive days. During the challenge period, the miR-770-5p-NC, miR-770-5p-antagomir, LV-NC, and LV-circ-0001454 treatment groups were administered interventions via tail vein injection at a concentration of 500 nmol/kg, three times per week.

### Airway hyperresponsiveness (AHR)

Airway responsiveness was measured within 24 h after the final HDM challenge. Methacholine, a commonly used agonist to induce airway constriction, was administered at increasing concentrations. Mice from each group were placed in a whole-body plethysmography chamber maintained at atmospheric pressure and exposed to nebulized Methacholine with stepwise increases in concentration (ranging from 0 mg/mL to 50 mg/mL). Measurements were recorded every 3 min, followed by an increase in Methacholine concentration. Lung resistance was expressed as Penh values, and changes in airway resistance were determined by measuring and recording pressure variations within the chamber, generating respiratory curves for each group.

### Lung injury score and lung coefficient

Assessment of Lung Injury: The lung tissue sections stained with hematoxylin and eosin (HE) were evaluated for pathological lung injury using a scoring system as follows: 0 points: Normal alveolar structure with no inflammatory cell infiltration. 1 point: Normal alveolar structure with mild injury (<25%) and minimal inflammatory cell infiltration. 2 points: Alveolar collapse with moderate injury (25%–50%) and limited inflammatory cell infiltration. 3 points: Loss of alveolar structure with severe injury (>50%) and extensive inflammatory cell infiltration. The scores from the four criteria were summed to obtain a total score, with higher total scores indicating more severe lung injury (Narasaraju et al., 2010).

Lung Index: Fresh and intact lung tissues from treated mice were rinsed repeatedly with PBS, and surface moisture was absorbed using filter paper. The total lung weight was measured, and the lung index was calculated as: Lung Index = (Lung Weight/Body Weight) × 100%.

### Cell culture and processing

Normal human bronchial epithelial cells (BEAS-2B) were purchased from the Cell Resource Center of the Shanghai Institute of Biological Sciences, Chinese Academy of Sciences (Shanghai, China). The cells were cultured in DMEM medium (C3110-0500, VivaCell, Shanghai, China) supplemented with 10% fetal bovine serum (c04001-500, VivaCell), 100 μg/mL streptomycin, and 100 U/mL penicillin (C3420-0100, VivaCell) at 37°C under 5% CO2. To stimulate the cells, they were treated with 5 μg/mL HDM (XPB46D3A4, Greer Laboratories, USA) for 24 h. When the cells reached 60% confluence, transfection was performed using Lipofectamine 3000 (L3000015; Thermo Scientific, USA), and cells were harvested 48 h post-transfection. Based on the transfection sequences, the experiments were divided into the following groups: HDM, vector+HDM, circ#1 (circ-0001454)+HDM, circ#2 (circ-0001454)+HDM, circ-NC+mimic-NC, circ-0001454+mimic-NC, circ-0001454+mimic-miR-770-5p, and circ-NC+mimic-miR-770-5p, with the control group receiving no treatment. All mimics and plasmids were purchased from RiboBio Co., Ltd. (Guangzhou, China).

### Lung tissue section staining

In the experimental protocol, left lung tissues were preserved using a 4% paraformaldehyde solution (P1110-, Solarbio, Beijing, China) for a duration of 1 week to ensure proper fixation. Following this preservation step, the tissues were embedded in paraffin and sectioned into 5 μm slices aimed for detailed pathological examination. These tissue sections were subsequently stained using HE (G1120, Solarbio, China), following standardized procedures for inflammation assessment as outlined in references (Mebratu et al., 2016; Crowe et al., 2009). In addition to HE staining, Masson’s trichrome staining technique (G1346, Solarbio, China) was employed to analyze collagen fiber deposition within the tissue structures. PAS staining (G1281, Solarbio, China) was also applied to specifically evaluate the proliferation and mucus secretion activities of goblet cells located around the airways. For comprehensive image analysis, a high-resolution slide scanning system (Model SQS-40R) from Shengqiang Technology Co., Ltd., based in Shenzhen, China, was utilized to capture and analyze the stained sections.

### Immunofluorescence staining

The protocol began by placing the prepared tissue sections in an oven set at a steady temperature of 60°C to effectively dissolve the paraffin wax in which they were embedded. Following this initial melting phase, the sections were subjected to a comprehensive dewaxing process utilizing xylene, and then sequentially treated with a series of ethanol solutions of varying concentrations to achieve thorough dehydration. For the purpose of antigen retrieval, microwave heating was employed, which facilitated the effective exposure of epitopes. This was followed by a critical step where the sections were incubated overnight with a-SMAantibody (14-9760-82,Thermo Fisher, China). After a thorough washing to remove any unbound antibodies, the tissue sections were subsequently incubated with a fluorescent-tagged secondary antibody-Goat anti-Mouse IgG H&L (HRP) (ab150113, Abcam, UK). To enhance the visualization of nuclei, the sections were counterstained with DAPI. The final step involved detailed imaging using a Cytation 5 fluorescence microscope, which allowed for the precise observation and documentation of cellular features and structures.

### Immunohistochemistry staining

The procedure initiated with the lung tissue sections being heated in an oven at 60°C to effectively melt the paraffin wax. Following this, the sections underwent a sequential dewaxing process using xylene, after which they were treated with a series of ethanol washes of ascending concentrations to ensure complete dehydration. After the dewaxing steps, the slides were subjected to microwave irradiation to facilitate optimal antigen retrieval, followed by a cooling phase in a buffered solution. Subsequent washing prepared the sections for the next phase of processing. They were then treated at room temperature with an endogenous peroxidase blocker to inhibit any unwanted enzymatic activity. The sections were further processed by incubating them with an α-SMA antibody (19245S,CST,USA), followed by application of a secondary antibody and DAB staining (ZLI-9018,PV-9000, ZSGB-BIO, China) to visualize the targeted proteins. Hematoxylin was used for counterstaining to enhance nuclear detail, after which the slides were again dehydrated through a graded series of ethanol, cleared with xylene, and finally mounted with neutral gum. High-resolution images of these sections were captured using a slide scanner (Model SQS-40R, Shengqiang Technology Co., China) and analyzed meticulously with ImageJ software to extract and quantify relevant cellular details.

### Collection of BALF

After euthanizing the mice by ether inhalation anesthesia, they were secured on a dedicated dissection board. The neck tissue was dissected to fully expose the trachea, and a small incision was made to gently insert and fix a fine catheter from an indwelling needle. Pre-cooled saline (1 mL per injection) was then slowly infused into the trachea while simultaneously massaging the thoracic cavity. Bronchoalveolar lavage fluid (BALF) was collected during the massage process. The procedure was considered successful if the BALF recovery rate reached 80% of the total saline infused, i.e., 0.8 mL.

### Counting of inflammatory factors in BALF

After the centrifugation process, the sediment from the BALF was resuspended in 1 mL of physiological saline. This step ensures that the cells are evenly dispersed for further analysis. Using a centrifuge, slides were prepared from this cell suspension, providing a uniform layer of cells for staining. The cell smears on the slides were then subjected to rapid staining using the Diff-Quik method (G540, Solarbio, China). This particular staining technique allows for the efficient differentiation and accurate counting of various cell types when examined under different magnifications in a microscope.

### Detection of pro-inflammatory cytokines in BALF

Quantitative measurements of the cytokines IL-4, IL-5, IL-13, and IFN-γ in BALF were conducted using a specific ELISA Kit (ZSGB-BIO, China). The assay was meticulously carried out in strict accordance with the detailed procedural instructions issued by the manufacturer to ensure accuracy and reliability of the results.

### Western blot

The standardized protocol for extracting and quantifying proteins from cellular structures and lung tissues includes the use of a nanophotometer NP80® (Implen, Bavaria, Munich, Germany) to accurately measure protein concentrations. This precision instrument ensures consistent results across experiments. Following concentration assessment, exactly 20 μg of protein per well is loaded onto SDS-PAGE gels for electrophoretic separation. This step is crucial for resolving the proteins based on their molecular weights. After electrophoresis, the proteins are carefully transferred to PVDF membranes, a process essential for subsequent detection steps. To ensure optimal antibody binding and minimize non-specific interactions, the membranes are blocked using non-fat milk. This step is followed by the incubation of the primary antibodies at 4°C overnight, a condition that enhances the binding specificity and efficiency. The primary antibodies used target a diverse range of proteins: β-actin (3700S, CST, United States), GAPDH (ab8245, Abcam, UK), Bax (2772, CST, United States), bcl-2 (17509, CST, United States), Cleaved-caspase-3 (9664, CST, United States), Cleaved-caspase-9 (20750S, CST, United States), α-SMA (19245S, CST, United States), NLRP3 (A12694, abclonal, China), IL-1β (12242s, CST, United States), p-AKT1 (9018, CST, United States), AKT1 (2938, CST, United States), MAPK1 (A17291, ABclonal, China), TGF-β1 (ab31013, Abcam, United Kingdom), cbl-b (sc-8006, Santa Cruz, United States), p-Smad3 (9145, CST, United States), and Lamin B1 (13435, CST, United States). Post-primary antibody incubation, a secondary antibody incubation at room temperature for one hour is performed. This secondary antibody, conjugated with horseradish peroxidase (HRP), facilitates the detection of the primary antibodies bound to the target proteins. Detection is achieved through the TECL chemiluminescence method (S0500, Millipore, United States), and the proteins are visualized using the AI600 gel imaging system (Bio-Tek, United States). This sophisticated imaging equipment allows for detailed analysis of the protein bands. Finally, the quantification of the proteins’ gray values is conducted using ImageJ software, providing a digital measure of protein abundance. The secondary antibodies employed include Goat anti-Rabbit IgG H&L (HRP) (ab205718, Abcam, Uited Kingdom) and Goat anti-Mouse IgG H&L (HRP) (ab205719, Abcam, United Kingdom), ensuring comprehensive detection across various immunoblotting applications.

### Dual-luciferase reporter assay analysis

Using the online resources circBank (https://www.circbank.cn/)and Circular RNA Interactome (https://www.circinteractome.irp.nia.nih.gov), potential binding sites were mapped out for the interaction between circ-0001454 and miR-770-5p, as well as between miR-770-5p and the gene cbl-b. To experimentally validate these interactions, both the wild type version of circ-0001454 (circ-0001454-WT) and a mutated variant (circ-0001454-Mut) were each transfected at 100 ng into BEAS-2B cells. This transfection process also included the introduction of 100 nM of either a miR-770-5p mimic or a non-coding mimic (mimic-NC) using Lipofectamine 3000 (L3000001, Invitrogen, United States). Following a 48-h incubation period under standard cell culture conditions, the effects of miR-770-5p mimic interaction with the circRNA constructs were quantified. Luciferase activity measured with dual-reporter assay kit (Promega).

### Fluorescence in situ hybridization (FISH) and nucleolar separation

The cells were first fixed in a solution of 4% paraformaldehyde, subsequently permeabilized with a chilled 0.5% solution of Triton X-100 to allow for probe access. For the detection of specific RNA sequences, hybridization was carried out using probes that were fluorescently labeled: circ-0001454 was tagged with Cy3, and miR-770-5p with FAM. To facilitate the visualization of nuclei, DAPI staining was applied, and observations of these stained samples were conducted using an advanced inverted fluorescence microscope, the Cytation 5 (BioTek, United States). The specific sequences of the probes utilized in these experiments are provided in Supplementary Table S1 of the supplementary materials. Furthermore, to separate nuclear components effectively, the PARIS™ kit (AM1921, Thermo Fisher Scientific, United States) was employed, following the manufacturer’s guidelines for optimal separation efficiency.

### Anti-agorna immunoprecipitation (RIP) assay

RIP assay used Millipore’s kit with RIP lysis buffer containing protease and RNase inhibitors. Lysates centrifuged, then incubated overnight at 4°C with magnetic beads conjugated to anti-Ago2 or IgG antibodies. This step facilitates the selective enrichment of RNA molecules associated with the Ago2 protein, which are critical for RNA-induced silencing complexes.

### Real-time PCR

Isolation of total RNA from the specified cells used Trizol reagent kit followed by manufactures guild lines. It was also accurately assessed how much the RNA was concentrated and of what purity. A reaction mixture, based on RNA measurements, was made using total RNA extracted from it as template. This mixture was used for the synthesis of cDNA: Using strict manufacturer’s protocols, miRNA and mRNA were reverse transcribed using the TaqMan® MicroRNA RT kit and a standard reverse transcription kit, respectively. The synthesised cDNA was quantified using the 2^−ΔΔCT^ method for detailed quantification of gene expression levels.

The sequences of primers used in our study are listed below: has-circ-0001454 forward:5′-GACAGCCGCATCTTCTTGTG-3′,reverse:5′-AATCCGTTCACACCGACCTT-3′; has-miR-770-5p forward:5′-UCCAGUACCACGUGUCAGGGCCA-3′; mmu-miR-770-5p forward:5′-AGCACCACGUGUCUGGGCCACG-3′; has-cbl-b forward:5′-CCGGTTAAGTTGCACTCGAT-3′,reverse:5′-CAAAGGGGTCCACGATTATG-3′; has-GAPDH forward:5′-GACAGCCGCATCTTCTTGTG-3′,reverse:5′-AATCCGTTCACACCGACCTT-3′; U6 forward:5′-GCTTCGGCAGCACATATACTAAAAT-3′,reverse:5′-GCTTCGGCAGCACATATACTAAAAT-3′; has-IL-6 forward:5′-ACTCACCTCTTCAGAACGAATTG-3′,reverse:5′-CCATCTTTGGAAGGTTCAGGTTG; has-IL-8 forward:5′-TTTTGCCAAGGAGTGCTAAAGA-3′; reverse:5′-AACCCTCTGCACCCAGTTTTC-3′,has-VEGF forward:5′-AGGGCAGAATCATCACGAAGT-3′,reverse:5′-AGGGTCTCGATTGGATGGCA-3′.The reverse primers of miR-770-5p were provided by matching the first strand cDNA synthesis kit of miRNAs tail method (B532451).

### Detection of reactive oxygen species

ROS was assayed in cellular environments with the application of DCFH-DA fluorescent probe (S0033S, Beyotime, China). The cells were incubated for 10 min at 37°C with this probe. Fluorescence, indicative of ROS levels, was subsequently detected using a Cytation 5 imaging system (BioTek, United States) and analysed with a flow cytometry on a Beckman Coulter device (United States). Detection of ROS in lung tissue samples was also performed similarly using dihydroethidium fluorescent probe (S0063, Beyotime, China). This probe was incubated with tissue slices for 5 min at 37°C, with visualisation and data capture using Cytation 5 system (BioTek, United States) for recording of fluorescence changes reflecting ROS activity within the tissues.

### Detection of mitochondrial membrane potential

The preparation of the JC-1 working solution (C2006, Beyotime, China) involved diluting the concentrated JC-1 stock to achieve a ratio of 1:The medium was 10 with the cell culture. These cells were also treated with 500 µL of diluted JC-1 solution and allowed to fully integrate before disposal of the top layer of media. The cells were then placed inside a 37°C room for 20 min allowing the staining of mitochondria within the cells. Fluorescence detection was performed post the incubation period using the Cytation 5 imaging system (BioTek, United States). Quantitative analysis of the red to green fluorescence intensity ratio of indicate mitochondrial membrane potential was performed using ImageJ software. A red-green fluorescence ratio wherein red decreases, and green remains the same represents decrease of mitochondrial membrane potential and change of mitochondrial functionality.

### TUNEL

Apoptosis in cell populations was then assessed using Annexin-V/7AAD staining protocol. I started with the process by bringing the cellular density to 1 × 10^6^ cells per millilitre followed by a thorough PBS wash. The cells then underwent digestion and centrifugation according to standard procedures, and were resuspended in 100 μL of Annenxin V binding buffer. To stain cells, 5 μL based on Annexin-V and 5 μL of 7AAD were added to this prepared suspension. Samples were gently mixed and stored in a dark environment at room temperature for 15 min to allow time for optimal interaction between components in the stain and in the cells. Subsequently, the stained cells were subjected to a sophisticated flow cytometry technique offered by Beckman (Coulter, United States). Herein, a method is described that enabled precise measurement of the apoptotic cell fraction as a function of the red and green fluorescence emitted, which is directly correlated to apoptotic status of the cells.

Cells were seeded in 12-well plates and washed once with PBS, followed by fixation with 4% paraformaldehyde for 30 min. After fixation, the cells were washed once with PBS. Subsequently, immunostaining strong permeabilization buffer (P0097, Beyotime, China) was added and incubated at room temperature for 5 min, followed by another PBS wash. Endogenous peroxidase blocking solution (P0100A, Beyotime, China) was then added to inactivate endogenous peroxidase activity, and the cells were washed three times with PBS. Biotin labeling solution and DAB chromogenic solution were prepared according to the manufacturer’s instructions. Fifty microliters of Streptavidin-HRP working solution was added to the samples and incubated at room temperature for 30 min, followed by three washes with PBS. Finally, 0.5 mL of DAB chromogenic solution was added and incubated at room temperature for 25 min. Stained samples were directly observed under a microscope.

### Experiment on actinomycin D

After preparing a solution of actinomycin D to a concentration of 2 g/L and incubating the actinomycin D solution in the cell culture incubator for various time points (0 h, 4 h, 8 h, 12 h, 24 h), the drug solution was used for performance of the experiments. However, after each responding duration, cells were harvested to collect total RNA and measure changes in gene expression using RT-qPCR.

### MTT cell viability assay

BEAS-2B cells were cultured at a density of 1 × 10^4^ cells per well within a 96-well plate. Following cell seeding, each well was supplemented with MTT reagent, prepared at a concentration of 0.5 mg/mL (C0009S, Beyotime, China). Subsequent experimental steps were undertaken according to the detailed protocol provided with the assay kit, ensuring that the process adhered strictly to the manufacturer’s guidelines for optimal results. Cell viability (%) = (OD value of cells treated with HDM/OD value of control cells) × 100%.

### Statistical analysis

Data analyzed with SPSS 19.0; ANOVA and the Q-test used for statistical evaluations, significance at *P* < 0.05 (mean ± SD).

## Results

### Inhibition of miR-770-5p can alleviate airway inflammation and airway remodeling in asthmatic mice

**FIGURE 1:**
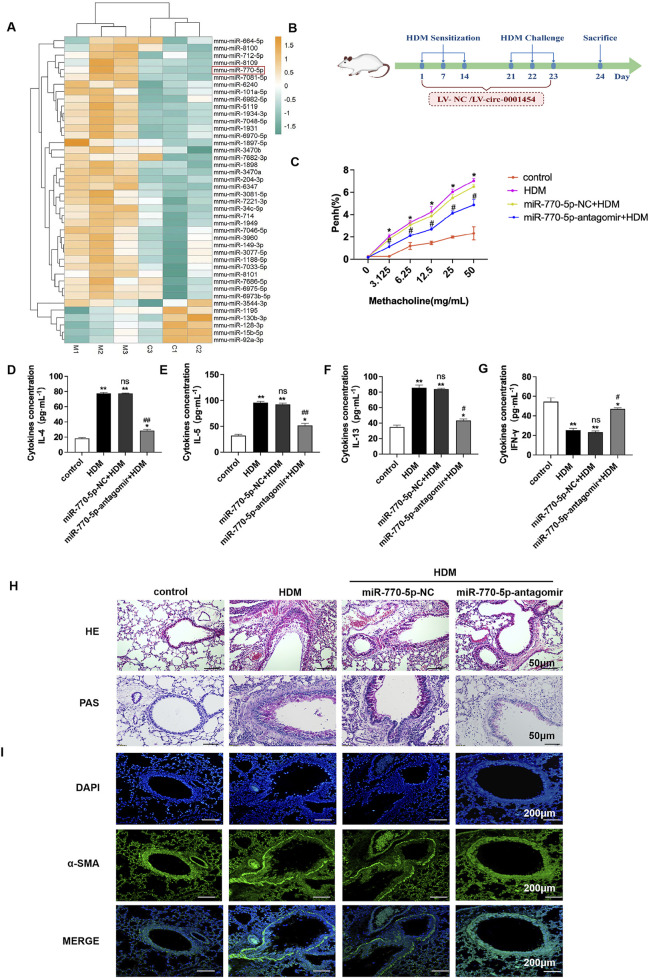
Inhibition of miR-770-5p can alleviate airway inflammation in asthmatic mice. **(A)** Analysis of the differential expression of microRNAs in asthmatic mice of microarray data sourced from the GEO database (accession number: GSE197090) involved hierarchical clustering of differentially expressed mRNAs using the R package pheatmap (version 1.0.12). **(B)** Illustration of the experimental setup for the HDM-induced asthma model treated with miR-770-5p antagomir (sample size: *n* = 6). **(C)** Measurement of airway hyperreactivity in treated mice (sample size: *n* = 6). **(D–G)** Quantification of cytokines IL-4, IL-5, IL-13, and IFN-γ in BALF via ELISA. **(H)** HE and PAS staining of lung tissue sections to assess histological changes (sample size: *n = 6*), with a scale of 50 µm. **(I)** Immunofluorescence staining of lung tissue sections (sample size: *n* = 6), scale set at 200 µm. Statistical significance: ^*^ *P* < 0.05, ^**^ *P* < 0.01 vs control; ^#^ *P* < 0.05, ^##^ *P* < 0.01 vs HDM; n.s. for non-significant.

To elucidate the involvement of miRNA in asthma’s pathogenesis, a gene chip analysis was performed, sourced from the following database. This analysis produced a heat map (Figure 1A) that highlighted miR-770-5p as significantly overexpressed in the experimental asthma model. Elevated expression of miR-770-5p was observed in asthmatic mice stimulated by house dust mites (HDM) compared with untreated controls (Supplementary Figure S1A).To rigorously test the regulatory role of miR-770-5p in asthma-related inflammation, an *in vivo* asthma model was developed using house dust mite (HDM) induction (Figure 1B). Airway hyperreactivity was subsequently quantified across various groups, revealing a notable reduction in the mice treated with an antagomir targeting miR-770-5p (Figure 1C.). Further investigations included ELISA tests to measure the concentrations of cytokines IL-4, IL-5, IL-13, and IFN-γ in bronchoalveolar lavage fluid (BALF). These tests showed that treatment with the miR-770-5p antagomir significantly lowered the levels of Th2-type inflammatory cytokines, while IFN-γ levels saw an increase (Figures 1D–G). Histology shows miR-770-5p antagomir reduces inflammation, hyperplasia, α-SMA, and remodeling in asthmatic mice (Figures 1H, I). Collectively, these results support the therapeutic potential of miR-770-5p inhibition in reducing airway inflammation and remodeling in models of asthma.

### Targeting circ-0001454 negatively regulates the expression of miR-770-5p

**FIGURE 2:**
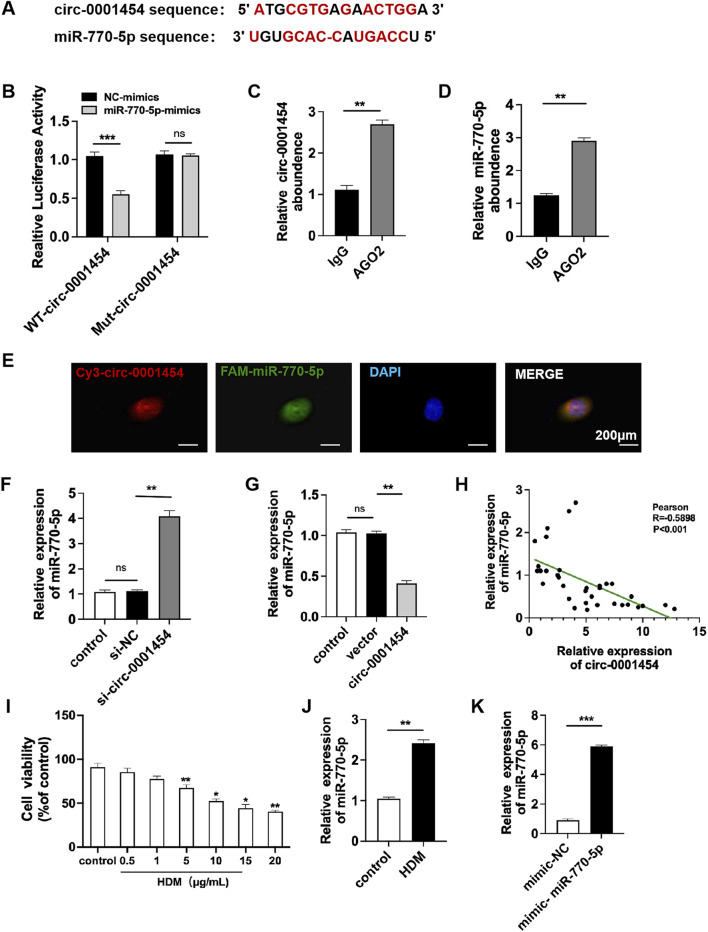
Targeting circ-0001454 negatively regulates the expression of miR-770-5p. **(A)** Predicted binding sites between circ-0001454 and miR-770-5p **.(B)** Results from dual-luciferase reporter assays. **(C–D)** Data from RNA immunoprecipitation experiments. **(E)** FISH analysis demonstrating co-localization of circ-0001454 and miR-770-5p in the cytoplasm of BEAS-2B cells, scale: 200 µm. **(F, G)** qPCR measures circ-0001454, miR-770-5p in BEAS-2B cells. **(H)** Pearson analysis of their relationship. **(I)** Investigation of the impact of various concentrations of HDM on cellular viability. **(J)** HDM induces higher miR-770-5p. **(K)** qPCR checks miR-770-5p transfection efficiency. ^*^ *P* < 0.05, ^**^ *P* < 0.01, ^***^ *P* < 0.001.

Research shows circ-0001454 decrease in asthmatic mice (Bao et al., 2020). miR-770-5p targeted via circBank (http://www.circbank.cn/) and Circular RNA Interactome (https://circinteractome.nia.nih.gov/) (Figure 2A). Confirmatory dual-luciferase reporter gene assays substantiated that circ-0001454 robustly binds to miR-770-5p, indicating a direct interaction (Figure 2B). To examine if circ-0001454 functions as a molecular sponge by sequestering miR-770-5p and influencing ceRNA mechanisms, RNA immunoprecipitation studies were performed. The results from these studies showed that circ-0001454 and miR-770-5p were both significantly enriched in the AGO2 complex relative to the control IgG in BEAS-2B cells, suggesting that circ-0001454 effectively absorbs miR-770-5p and inhibits its function (Figures 2C, D). Fluorescence *in situ* hybridization (FISH) was employed to visually confirm the co-localization of circ-0001454 and miR-770-5p within the cytoplasm, illustrating their interaction at a cellular level (Figure 2E). Quantitative PCR analyses further quantified the impact of this interaction on the expression levels of both RNA molecules (Figures 2F, G). Pearson correlation analysis demonstrated a significant inverse relationship between the expression of circ-0001454 and miR-770-5p (Figure 2H). Subsequent to the transfection, the BEAS-2B cells underwent treatment with varying concentrations of HDM allergen (0.5, 1, 5, 10, 15, 20 μg/mL) diluted in DMEM over a 24-h period. MTT assay: HDM reduces BEAS-2B cell viability at 5 μg/mL (Figure 2I). In response to stimulation with house dust mite (HDM), an increase in miR-770-5p expression was observed in BEAS-2B cells compared to untreated controls, underscoring the responsiveness of miR-770-5p to inflammatory stimuli (Figure 2J). To facilitate the exploration of this regulatory effect, a miR-770-5p mimic and its corresponding control vector were engineered. Quantitative PCR confirmed the high efficiency of transfection, ensuring that the mimic was adequately expressed within the cells (Figure 2K). Collectively, these findings indicate that circ-0001454 acts as a sponge, absorbing miR-770-5p and thereby modulating its availability to regulate gene expression negatively. This mechanism suggests a novel therapeutic target for managing and understanding the molecular basis of asthma.

### Screening and Identification of circ-0001454

**FIGURE 3:**
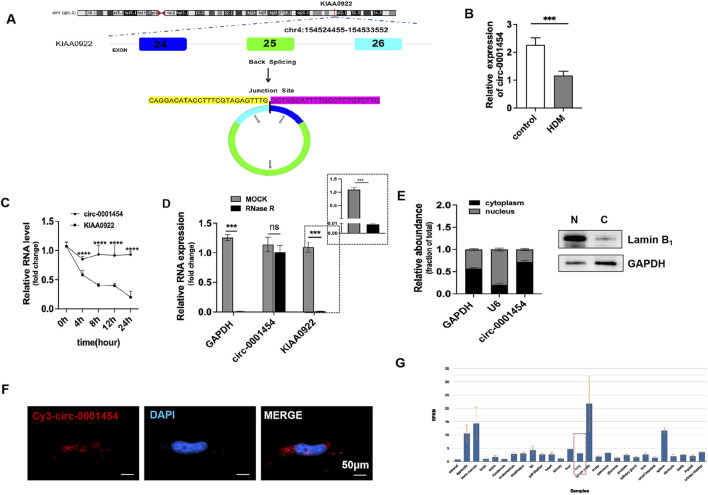
Screening and Identification of circ-0001454. **(A)** Discovery of circ-0001454. **(B)** Expression of IRC-0001454 in inflammatory conditions. **(C)** Examination of circ-0001454 and KIAA0922 mRNA stability in BEAS-2B cells treated with actinomycin D. **(D)** Stability analysis of circ-0001454 and KIAA0922 mRNA post-RNase R treatment in BEAS-2B cells. **(E)** Localization of circ-0001454 in BEAS-2B cells assessed by FISH, displayed at a scale of 200 µm. **(F)** Subcellular distribution of circ-0001454 within BEAS-2B cells. **(G)** Distribution details of circ-0001454 sourced from the NCBI database. Significant statistical values indicated by ^***^ *P* < 0.001, ^****^ *P* < 0.001.

Previous investigations have indicated a significant reduction in the expression of circ-0001454 in models of asthma (Bao et al., 2020). Comprehensive database searches within the UCSC Genome Database (http://genome.ucsc.edu) and circBase(http://www.circbase.org) identified that the circular RNA molecule, hsa-circ-0001454, originates from exons 24 to 26 of the NM_001131007 transcript from the parent gene KIAA0922, located on chromosome 4 in the region chr4:154524455–154533552. This RNA forms a circular structure by back-splicing across a genomic distance of 9098 base pairs. The specific site of circularization, confirmed by CircPrimer software, is located at the splice junction between sequences TTTG and GCTA (Figure 3A). To quantify the expression changes of circ-0001454 under inflammatory conditions, qRT-PCR assays were performed after treating BEAS-2B cells with house dust mite (HDM), revealing a notable decrease in circ-0001454 levels (Figure 3B). The molecular stability of circ-0001454 was further assessed through experiments using actinomycin D and resistance to enzymatic degradation. RT-qPCR analysis following actinomycin D treatment showed that while the mRNA levels of the parental gene KIAA0922 significantly diminished, the levels of circ-0001454 exhibited minimal alterations, indicating its increased stability and prolonged half-life as a circular RNA (Figure 3C). Additional experiments involving RNase R treatment in BEAS-2B cells demonstrated a significant reduction in the linear mRNA levels of KIAA0922, whereas the circular form, circ-0001454, remained stable, reinforcing its structural integrity and resistance to RNase R degradation (Figure 3D). Localization studies using nucleus-cytoplasm separation techniques and FISH confirmed that circ-0001454 predominantly localizes in the cytoplasm of BEAS-2B cells (Figures 3E, F). Data from the NCBI database further corroborate the expression of circ-0001454 specifically in lung tissues, suggesting its functional relevance in pulmonary biology (Figure 3G). These findings collectively enhance our understanding of the stability and cellular localization of circ-0001454, highlighting its potential role as a regulatory RNA in the pathophysiology of asthma and possibly other respiratory conditions.

### Up-regulation of circ-0001454 alleviates inflammatory injury of bronchial epithelial cells

**FIGURE 4:**
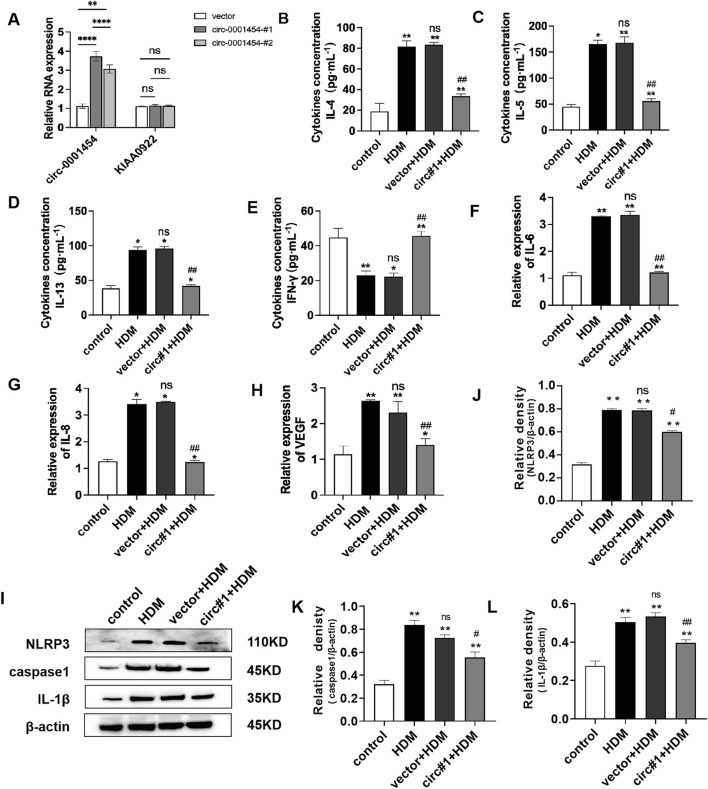
Upregulation of circ-0001454 alleviates inflammatory injury of bronchial epithelial cells. **(A)** Elevated expression of circ-0001454 and its host gene KIAA0922 was assessed. **(B–E)** Levels of inflammatory cytokines IL-4, IL-5, IL-13, and IFN-γ were quantified using ELISA. **(F–H)** Quantitative PCR was utilized to measure mRNA levels of IL-6, IL-8 and VEGF, **(I-L)** Induction of NLRP3 activation following overexpression of circ-0001454. Statistical significance indicated by ^*^ *P* < 0.05, ^**^ *P* < 0.01 compared to the control group, ^*##*^ *P <* 0.01, n.s, compared to the HDM group.

To assess the impact of circ-0001454 on the inflammatory damage within bronchial epithelial cells, overexpression plasmids targeting circ-0001454 were transfected into the BEAS-2B cell line. The efficacy of this transfection was verified through quantitative PCR, which indicated a substantial elevation in the expression of circ-0001454 without a corresponding increase in the expression of its parental gene, KIAA0922. This led to the selection of the construct designated as circ-0001454#1, which demonstrated the highest transfection efficiency, for all further experimental activities (Figure 4A). Consequently, a 5 μg/mL concentration of HDM was selected to stimulate the cells for subsequent assays. Further analyses were conducted to understand the effects of circ-0001454 overexpression on cytokine production. Both ELISA and quantitative PCR were employed to measure the levels of IL-4, IL-5, IL-13, and IFN-γ, as well as the mRNA expression levels of inflammatory markers VEGF, IL-6, and IL-8 in the BEAS-2B cells. The ELISA outcomes revealed that the overexpression of circ-0001454 markedly suppressed the levels of HDM-induced cytokines such as IL-4, IL-5, and IL-13, while simultaneously enhancing the level of IFN-γ (Figures 4B–E). Additionally, the quantitative PCR results demonstrated that the mRNA expression levels of VEGF, IL-6, and IL-8 were significantly reduced in the circ-0001454 transfection group in comparison to the group treated with HDM alone (Figures 4F–H). Further investigation was conducted to determine whether overexpression of circ-0001454 could inhibit the activation of the NLRP3 inflammasome and the synthesis of IL-1β in the bronchial epithelial cells. Western blot analyses were performed to detect the expression of NLRP3, IL-1β, and caspase-1. These analyses indicated that the levels of these inflammatory mediators were reduced following the overexpression of circ-0001454 (Figures 4I–L). These comprehensive results suggest that upregulation of circ-0001454 can modulate the HDM-induced inflammatory response in BEAS-2B cells, effectively alleviating inflammatory damage within bronchial epithelial cells. This underscores the therapeutic potential of circ-0001454 as a modulator of inflammatory pathways in respiratory conditions.

### circ-0001454 upregulation reduce bronchial epithelial cell oxidative stress, apoptosis and mitochondrial membrane potential loss

**FIGURE 5:**
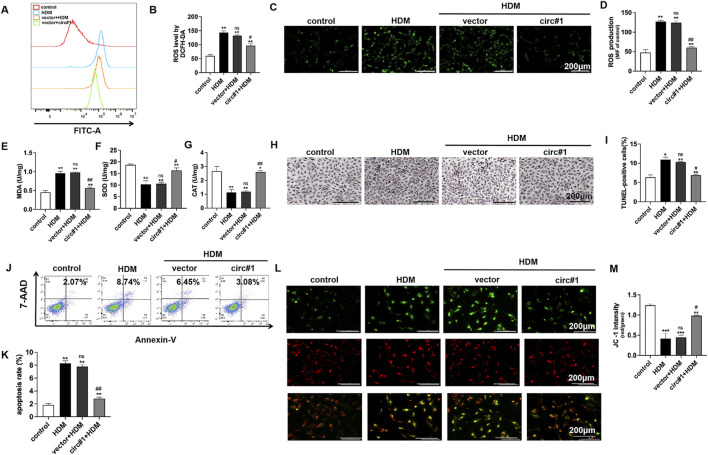
circ-0001454 upregulation reduce bronchial epithelial cell oxidative stress, apoptosis and mitochondrial membrane potential loss. **(A–B)** Assessment of ROS levels in BEAS-2B cells via flow cytometry. **(C–D)** Immunofluorescence staining to visualize ROS accumulation in BEAS-2B cells, scale bar: 200 µm. **(E-G)** ELISA employed to measure the levels of antioxidant enzymes SOD, CAT, and MDA in BEAS-2B cells. **(H–I)** TUNEL staining to quantify apoptosis in BEAS-2B cells. **(J–K)** Apoptosis quantification in BEAS-2B cells using flow cytometry. **(L–M)** JC-1 staining to evaluate changes in mitochondrial membrane potential, scale bar: 200 µm. Statistical significance denoted as ^*^ *P* < 0.05, ^**^ *P* < 0.01, ^***^ *P* < 0.001 versus control group; ^#^ *P* < 0.05, ^##^ *P* < 0.01, n.s. versus HDM group.

Flow cytometry was utilized to monitor changes in reactive oxygen species (ROS) in bronchial epithelial cells after overexpressing circ-0001454. The data indicated a notable decrease in the mean fluorescence intensity of ROS, highlighting a reduction in oxidative stress markers (Figures 5A, B). Further fluorescence analyses corroborated that ROS levels, initially elevated due to HDM stimulation, were markedly reduced following the introduction of the circ-0001454 overexpression plasmid (Figures 5C, D). The influence of circ-0001454 on antioxidant defense mechanisms within these cells was then quantitatively assessed using enzyme-linked immunosorbent assays. Transfection boosted SOD, CAT activities; reduced MDA levels (Figures 5E–G). Additional experiments included TUNEL staining combined with flow cytometry to evaluate the anti-apoptotic effects of circ-0001454 in these cells. Findings from these assays revealed that the upregulation of circ-0001454 significantly curtailed cell apoptosis, suggesting enhanced cellular resilience (Figures 5H–K). JC-1 staining was also performed to assess changes in mitochondrial membrane potential, which is often compromised during oxidative stress. The results demonstrated that the upregulation of circ-0001454 effectively restored the mitochondrial membrane potential disrupted by HDM exposure (Figure 5l, M). We also measured the ATP content of each group, and the results showed that circ-0001454 could restore the ATP level (Supplementary Figure S2A). Overall, these findings suggest that enhancing the expression of circ-0001454 in bronchial epithelial cells can substantially mitigate oxidative stress, reduce cellular apoptosis, and stabilize mitochondrial membrane potential, thereby underscoring its potential therapeutic value in treating respiratory epithelial cell damage induced by environmental stressors.

### circ-0001454 inhibits miR-770-5p expression and alleviates inflammatory injury of bronchial epithelial cells

**FIGURE 6:**
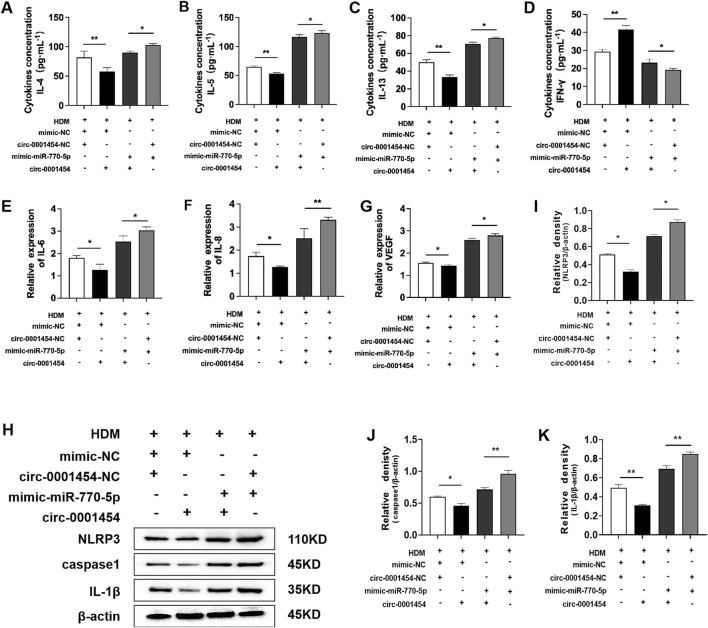
circ-0001454 inhibits the expression of miR-770-5p, alleviating inflammatory damage in bronchial epithelial cells. **(A–D)** Levels of cytokines IL-4, IL-5, IL-13, and IFN-γ were quantified using ELISA. **(E–G)** Expression levels of VEGF, IL-6, and IL-8 mRNA were assessed by qPCR. **(H–K)** Enhanced expression of circ-0001454 reduced NLRP3 activation and IL-1β synthesis. Statistical significance indicated by ^*^ *P* < 0.05, ^**^ *P* < 0.01.

To establish that circ-0001454 acts as a competing endogenous RNA (ceRNA) by specifically targeting miR-770-5p, we conducted a series of functional rescue assays. These included ELISA and quantitative PCR (qPCR) to evaluate the cytokine profiles within BEAS-2B cells post-transfection. Results from ELISA indicated a pronounced suppression of pro-inflammatory cytokines IL-4, IL-5, and IL-13, alongside an elevation in the anti-inflammatory cytokine IFN-γ, in cell lines that were stably overexpressing miR-770-5p but subsequently transfected with circ-0001454 (Figures 6A–D). qPCR shows IL-6, IL-8 and VEGF mRNA decrease with circ-0001454 (Figures 6E–G). Additionally, Western blot analysis was employed to examine the impact of circ-0001454 on the activation of the NLRP3 inflammasome and the synthesis of IL-1β within bronchial epithelial cells impacted by miR-770-5p. The results revealed that circ-0001454 effectively mitigated the activation of NLRP3 inflammasomes and reduced IL-1β production, further supporting the ceRNA mechanism of action (Figures 6H, I). Collectively, these results provide compelling evidence that circ-0001454 can downregulate miR-770-5p expression, thereby alleviating inflammatory damage in bronchial epithelial cells.

### circ-0001454 suppressed miR-770-5p expression, reducing oxidative stress, apoptosis and mitochondrial membrane potential loss in bronchial epithelial cells

**FIGURE 7:**
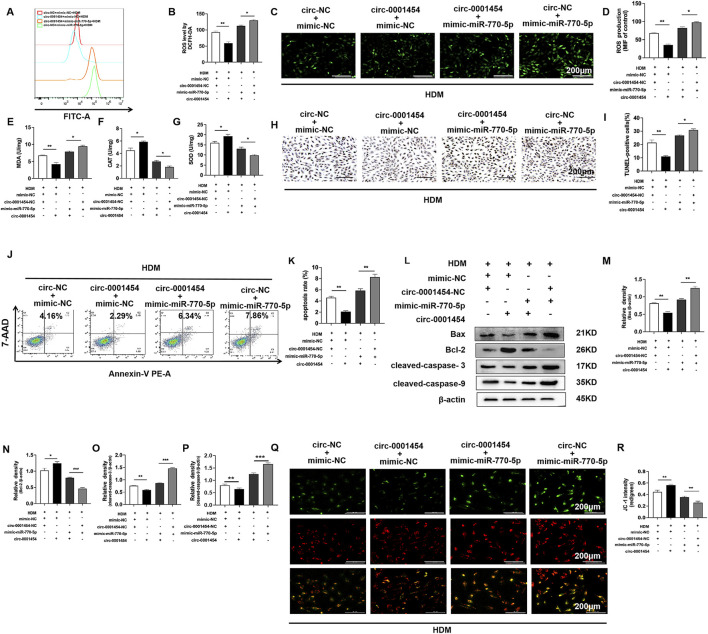
circ-0001454 inhibits the expression of miR-770-5p, reducing oxidative stress, apoptosis, and the loss of mitochondrial membrane potential in bronchial epithelial cells. **(A, B)** Assessment of ROS levels in BEAS-2B cells via flow cytometry. **(C, D)** Immunofluorescence used to evaluate ROS distribution in BEAS-2B cells, scale bar: 200 µm. **(E–G)** Levels of antioxidant enzymes SOD, CAT, and MDA quantified using ELISA. **(H, I)** TUNEL assay employed to quantify apoptosis in BEAS-2B cells, scale bar: 200 µm. **(J, K)** Flow cytometry used to measure apoptosis levels in BEAS-2B cells. **(L-P)** Western blot measures apoptosis proteins: Bax, Bcl-2, cleaved-caspases. **(Q, R)** JC-1 staining to assess mitochondrial membrane potential, scale bar: 200 µm. Significance indicated by ^*^ *P* < 0.05, ^**^ *P* < 0.01.

To further investigate the impact of circ-0001454 on the cellular dynamics affected by the overexpression of miR-770-5p, particularly focusing on oxidative stress, apoptosis, and mitochondrial dysfunction, we implemented a series of assays using flow cytometry and fluorescence staining techniques. These studies aimed to monitor the alterations in cellular reactive oxygen species (ROS) levels following the co-expression of circ-0001454 with miR-770-5p. The collected data revealed that the introduction of circ-0001454 alongside miR-770-5p significantly diminished ROS levels within the cells (Figures 7A–D). Concurrently, Increased SOD, CAT activity and reduced MDA indicate less oxidative stress (Figures 7E–G). Additionally, apoptosis analysis through TUNEL staining and subsequent flow cytometric assessment demonstrated that circ-0001454 has a potent ability to mitigate the increased apoptosis rates induced by miR-770-5p overexpression in bronchial epithelial cells (Figures 7H–K). Western blot: apoptosis proteins decrease, circ-0001454 inhibits apoptosis (Figures 7L–P). Furthermore, JC-1 staining was utilized to evaluate the mitochondrial membrane potential, which is often compromised in cells under stress. The results confirmed that the mitochondrial dysfunction observed upon miR-770-5p overexpression was effectively reversed post-transfection of circ-0001454, restoring normal mitochondrial function (Figures 7Q, R). At the same time, we found that circ-0001454 could restore ATP loss caused by miR-770-5p overexpression (Supplementary Figure S3A). Collectively, these findings underscore the multifaceted role of circ-0001454 in ameliorating oxidative stress, reducing apoptosis, and restoring mitochondrial stability by counteracting the effects of miR-770-5p in bronchial epithelial cells.

### circ-0001454 targeting miR-770-5p to upregulate cbl-b levels and alleviate airway inflammation

**FIGURE 8:**
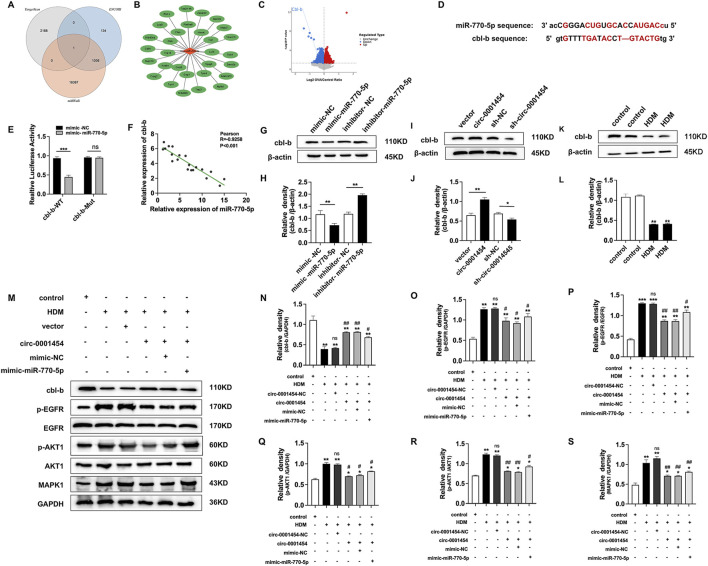
**c**irc-0001454 targets miR-770-5p to upregulate cbl-b levels and alleviate airway inflammation. **(A)** Bioinformatics platforms used to predict miR-770-5p target genes, illustrated through a Venn diagram. **(B)** Bioinformatics analysis employed to refine the screening of target genes. **(C)** Analysis of data sourced from the GEO database (accession number: GSE197090) involved Volcano map of differentially expressed cbl-b. **(D)** Predictive analysis of miR-770-5p′s binding sites on cbl-b. **(E)** Execution of dual-luciferase reporter assays to verify interactions. **(F)** Pearson correlation analysis elucidating the relationship between miR-770-5p and cbl-b. **(G–H)** Western blot assays to examine the correlation between miR-770-5p and cbl-b protein expression. **(I–J)** Western blot analysis to explore the association between circ-0001454 and cbl-b expression. **(K–L)** Detection of low cbl-b expression levels in HDM-induced BEAS-2B cells **.(M–S)** Conduct of functional rescue experiments to assess impact on airway inflammation. Statistical significance denoted by ^*^ *P* < 0.05, ^**^ *P* < 0.01, ^***^ *P* < 0.001 compared to control group; ^#^ *P* < 0.05, ^##^ *P* < 0.01, n.scompared to HDM group.

Leveraging bioinformatics resources such as TargetScan (https://www.targetscan.org/vert_80/), ENCORI (https://rnasysu.com/encori/),and miRWalk (http://mirwalk.umm.uni-heidelberg.de), we identified a singular potential target gene of miR-770-5p by analyzing the intersections with genes that were downregulated in our RNA-seq dataset (Figure 8A). This gene was selected for further analysis through rigorous bioinformatics screening processes (Figure 8B). In our database (accession number: GSE197090), the expression of cbl-b was lower in the OVA compared to the normal group (Figure 8C). Subsequent predictions from these databases indicated potential interaction sites between miR-770-5p and the gene cbl-b, suggesting a direct regulatory relationship (Figure 8D). In line with our computational forecasts, the dual-luciferase reporter assay substantiated the presence of these interaction sites, confirming the predictive accuracy of our bioinformatics approach (Figure 8E). Pearson analysis shows negative correlation between miR-770-5p, cbl-b (Figure 8F). This analysis provided a foundational understanding of their interactive dynamics. To further validate these findings, Western blot analyses were executed, which affirmed the inverse correlation between miR-770-5p and cbl-b expression levels, reinforcing the notion that miR-770-5p may be a critical regulator of cbl-b (Figures 8G, H). Exploring further, we also evaluated the relationship between circ-0001454 and cbl-b. Western blot analyses indicated a positive correlation between the expressions of circ-0001454 and cbl-b, suggesting that circ-0001454 may enhance cbl-b expression in bronchial epithelial cells (Figures 8I, J). Additionally, it was observed that cbl-b expression decreased under inflammatory conditions, highlighting its potential role in inflammatory responses (Figures 8K, L). To explore whether circ-0001454 has a direct regulatory effect, we conducted a Western blot analysis. The results showed that after adding the miR-770-5p inhibitor, circ-0001454 also had a regulatory effect on cbl-b (Supplementary Figures S4A, B). To dissect the regulatory mechanism further, functional rescue experiments were carried out to determine whether cbl-b expression is modulated by the circ-0001454/miR-770-5p axis. Western blot results demonstrated that transfection with circ-0001454 alone increased cbl-b protein expression following HDM induction. However, subsequent co-transfection with a miR-770-5p mimic reduced cbl-b protein levels, suggesting a modulatory effect of circ-0001454 on miR-770-5p′s regulation of cbl-b (Figures 8M–S). These findings confirm that circ-0001454 targets miR-770-5p and plays a significant role in regulating HDM-induced cbl-b levels, which in turn influences the expression of crucial signaling proteins such as EGFR, AKT1, and MAPK1, thereby mitigating inflammation in airway epithelial cells.

### Up-regulation of circ-0001454 alleviates airway inflammation in asthmatic mice and reduces airway remodeling

**FIGURE 9:**
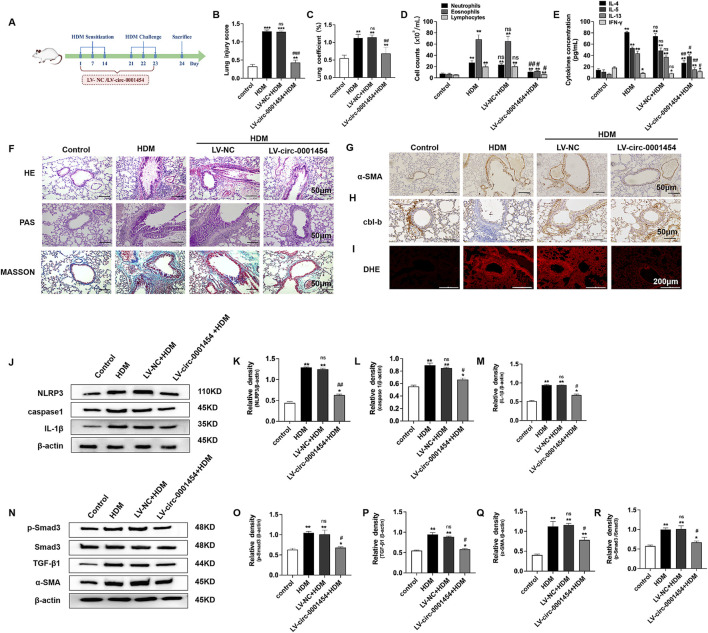
Upregulation of circ-0001454 alleviates airway inflammation in asthmatic mice. **(A)** Diagram of the HDM-induced asthma model and LV-circ-0001454 treatment approach (*n = 6*). **(B)** Assessment of lung injury scores in mice (*n = 6*). **(C)** Lung coefficients measured in experimental subjects (*n = 6*). **(D)** Diff-Quik staining for inflammatory cell analysis in BALF (*n = 6*). **(E)** ELISA-based cytokine levels (IL-4, IL-5, IL-13, and IFN-γ) in BALF. **(F)** Histological sections of lung tissue stained with HE, PAS, and Masson’s trichrome (*n = 6*), scale bar: 50 µm. **(G)** Immunohistochemical α-SMA detection in lung sections (*n = 6*), scale bar: 50 µm. **(H)** cbl-b levels in lung tissues through immunohistochemical methods (*n = 6*), scale bar: 50 µm. **(I)** Oxidative stress visualization in lung tissues via DHE staining (*n = 6*), scale bar: 200 µm. **(J–M)** Western blot analysis for caspase-1, NLRP3,and IL-1β in lungs of asthmatic mice. **(N–R)** TGF-β1, Smad3, and α-SMA protein levels in asthmatic mice’s lung tissues assessed by Western blot. Statistical indicators: ^*^ *P* < 0.05, ^**^ *P* < 0.01 versus control group, and ^#^ *P* < 0.05, ^##^ *P* < 0.01, n.s., versus HDM group.

To deepen our understanding of circ-0001454s role in modulating airway inflammation, we executed *in vivo* studies by establishing House Dust Mite (HDM)-induced asthma models in mice (Figure 9A). Enhancing the expression of circ-0001454 in these models demonstrated significant normalization of the lung injury scores and lung coefficients, suggesting a substantial reduction in tissue damage (Figures 9B, C). Further analysis involved Diff-Quik staining of bronchoalveolar lavage fluid (BALF) from each experimental group to quantify inflammatory cells. The results indicated a pronounced decrease in the counts of neutrophils, eosinophils, and lymphocytes in mice with elevated circ-0001454 levels, highlighting its potent anti-inflammatory effects (Figure 9D). Additionally, ELISA assays were conducted to measure the cytokine profiles in the BALF. Assays show cytokine changes in circ-0001454-overexpressed mice (Figure 9E). Pathological examinations of lung tissue sections from these asthmatic models were performed, showing that circ-0001454 upregulation diminished inflammatory cell infiltration around airways and blood vessels, reduced goblet cell hyperplasia, and decreased collagen deposition (Figure 9F). Immunohistochemical analyses corroborated these findings with a noted decrease in α-SMA expression (Figure 9G), and a reduction in cbl-b expression was also observed (Figure 9H). Immunofluorescence staining of the lung tissues further confirmed a decrease in ROS levels in mice with upregulated circ-0001454 (Figure 9I). Western blot analyses were carried out to assess the expression levels of NLRP3, IL-1β, and caspase-1. The results indicated that enhanced expression of circ-0001454 effectively inhibited the activation of the NLRP3 inflammasome and reduced the synthesis of IL-1β in the asthmatic mice (Figures 9J–M). Moreover, there was a notable reduction in the expression levels of TGF-β1, Smad3, and α-SMA, which are critical markers of airway remodeling (Figures 9N–R). Western blot analysis was performed to detect the protein expression levels of cbl-b and its regulated target genes in mice. We found that upregulation of circ-0001454 significantly increased the expression of cbl-b while decreasing the expression of its target genes compared to the HDM group (Supplementary Figure S5A–G).

These comprehensive findings demonstrate that circ-0001454 plays a crucial role in alleviating airway inflammation and remodeling in asthma models, potentially offering a therapeutic target for managing chronic respiratory conditions.

## Discussion

Bronchial asthma is recognized globally as a chronic inflammatory disorder of the airways that poses a significant health threat. Recent research underscores the crucial role of microRNAs (miRNAs) in the modulation of airway inflammation in asthma, where these miRNAs can either exacerbate or mitigate inflammation through diverse pathways. Notably, a study by Li et al. (2023) identified that miR-770-5p intensifies inflammation and oxidative stress in diabetic nephropathy mouse models, thereby aggravating renal damage. Parallel investigations in our laboratory have previously demonstrated a marked elevation in the expression of miR-770-5p in lung tissues of mice with asthma (Supplementary Figure S1A) (Xu et al., 2022). Building on these findings, we undertook comprehensive experiments to explore the specific influence of miR-770-5p on asthma pathology. Our experimental outcomes reveal that treatment of asthmatic mice with an antagomir targeting miR-770-5p led to a significant amelioration of airway hyperreactivity. This treatment also resulted in decreased concentrations of pro-inflammatory cytokines IL-4, IL-5, and IL-13, alongside an increase in the anti-inflammatory cytokine IFN-γ. Furthermore, there was a notable reduction in the infiltration of inflammatory cells around the airways and vascular structures, diminished goblet cell hyperplasia, and a decrease in the expression of α-SMA. Collectively, these findings strongly suggest that the inhibition of miR-770-5p could be a viable strategy to alleviate airway inflammation and remodeling in asthma, offering potential pathways for therapeutic intervention.

Further investigations have identified a potential interactive site between miR-770-5p and circ-0001454, suggesting a complex interaction. To elucidate whether circ-0001454 mediates its effects through the sequestration of miR-770-5p, we executed RNA immunoprecipitation experiments. These experiments demonstrated that both circ-0001454 and miR-770-5p are co-enriched with AGO2 protein, supporting the hypothesis that circ-0001454 functions as a molecular sponge, absorbing miR-770-5p and modulating its availability to influence ceRNA activities. This mechanism indicates that they are co-localized within the cytoplasmic environment. Considering the pivotal role that miRNAs play in the pathophysiological processes of airway allergic diseases, it becomes apparent that circRNAs like circ-0001454 could exert significant regulatory influence. Through their interactions with miRNAs, circRNAs can distinctly affect the inflammatory responses within bronchial epithelial cells, potentially offering novel therapeutic targets for managing airway inflammation (Bhat et al., 2024). Initial findings have particularly focused on circ-0001454 due to its reported low expression in models of asthmatic mice (Bao et al., 2020), highlighting its potential importance in respiratory inflammatory conditions.

To elucidate the specific function of circ-0001454 in the context of inflammatory processes, a series of *in vitro* and *in vivo* studies were initiated. These studies demonstrated that circ-0001454 is typically underexpressed during inflammatory responses. Elevating the levels of circ-0001454 in cellular models led to a notable mitigation of inflammation in bronchial epithelial cells, characterized by a decrease in IL-4, IL-5, IL-13 levels, and an increase in IFN-γ levels. Furthermore, this upregulation also resulted in the reduction of IL-6, IL-8, and VEGF mRNA levels, alongside effective suppression of NLRP3 inflammasome activation and IL-1β synthesis. Inflammatory and immune cells, particularly eosinophils, and neutrophils in the airways of asthmatic subjects, exhibit heightened activation and increased oxidative stress, contributing to cellular damage. This oxidative stress further exacerbates airway inflammation (Michaeloudes et al., 2022). Excessive ROS levels, when not adequately neutralized by intracellular antioxidants, can lead to a significant decrease in mitochondrial membrane potential and the release of apoptotic proteins (Bronner and O'Riordan, 2016). Research by Rahman et al. (Rahman et al., 1996) has indicated that MDA levels, a marker of oxidative stress, are elevated in asthmatic patients compared to healthy individuals, and are significantly higher during acute asthma attacks than in stable conditions. Additionally, it has been observed that SOD, a crucial antioxidant enzyme, exhibits reduced activity in newly diagnosed asthmatic patients compared to non-asthmatic controls (Yang et al., 2011). Our experiments revealed that enhancing circ-0001454 expression not only alleviated inflammatory damage but also diminished ROS expression levels and reduced CAT and MDA levels while increasing SOD activity. This adjustment also decreased cell apoptosis and stabilized mitochondrial transmembrane potential. And also restored the lost ATP (Supplementary Figure S2A). In animal models, augmenting circ-0001454 expression in asthmatic mice led to reductions in lung damage, lung coefficients, and the counts of neutrophils, eosinophils, and lymphocytes in bronchoalveolar lavage fluid. It also resulted in lowered levels of IL-4, IL-5, and IL-13, increased IFN-γ levels, reduced infiltration of inflammatory cells around the airways and blood vessels, diminished goblet cell hyperplasia, decreased α-SMA expression, and mitigated airway remodeling. Furthermore, upregulation of circ-0001454 also enhanced the expression of cbl-b in these models, impacting the expression of DHE in lung tissue. Studies have shown that reducing TGF-β1 expression and inhibiting SMAD3 phosphorylation may alleviate airway remodeling in asthma (He et al., 2021). Western blot results from these studies suggest that upregulation of circ-0001454 in asthmatic mice can effectively alleviate airway remodeling through these pathways.

The circular structure of circRNA is enriched with miRNA binding sites, enabling a single circRNA to adsorb dozens or even hundreds of miRNAs, thereby functioning as a molecular sponge. This mechanism competitively sequesters miRNAs that would otherwise bind to mRNA, thereby relieving miRNA-mediated repression of mRNA. This regulatory mechanism is known as the competing endogenous RNA (ceRNA) mechanism (Piecka et al., 2017; Weng et al., 2017). The role of circular RNAs, particularly in cellular localization, is pivotal for understanding their function. Circular RNAs located in the cytoplasm often function as microRNA sponges, facilitating ceRNA mechanisms (Tang et al., 2021). Through FISH and nuclear-cytoplasmic separation experiments, we determined that circ-0001454 predominantly resides in the cytoplasm. Previous RNA immunoprecipitation experiments substantiated that circ-0001454 can sequester miR-770-5p, thereby modulating its biological activity to perform ceRNA functions. In an extension of this research, functional rescue assays were conducted which demonstrated that enhancing circ-0001454 expression, in conjunction with miR-770-5p transfection, can ameliorate the inflammatory damage observed in airway epithelial cells induced by miR-770-5p. This confirms circ-0001454s role in mitigating inflammation by modulating miR-770-5p, contributing to the reduction of excessive ROS expression, curbing apoptosis, and stabilizing mitochondrial transmembrane potential. And also restored the lost ATP (Supplementary Figure S3A) miRNAs are known to bind the 3′ UTR regions of target gene mRNAs, influencing gene expression and regulating cellular processes such as proliferation, migration, and apoptosis (Eulalio et al., 2008). Using bioinformatics tools, we identified cbl-b as a potential target of miR-770-5p, which was confirmed through dual-luciferase reporter assays. The cbl-b is critical in the regulation of inflammation, particularly noted in studies of LPS-induced microglial cell inflammation, where reduced expression suppresses inflammation via PI3K/AKT pathway regulation (Dong et al., 2016). The cbl-b negatively regulates the TLR4 signaling pathway in monocytes in an acute lung injury model. Mice lacking cbl-b exhibited significantly aggravated lung inflammation and were prone to sepsis (Bachmaier et al., 2007). These findings suggest that cbl-b is involved in the regulation of lung inflammation and may play a role in the pathogenesis of asthma by modulating inflammatory responses. However, systematic studies on the mechanism linking cbl-b to asthma are currently lacking. Our findings also highlight that cbl-b expression is downregulated in HDM-induced inflammation but shows a positive correlation with circ-0001454 and a negative correlation with miR-770-5p. Under the interference of miR-770-5p inhibitors, circ-0001454 also has a regulatory effect on cbl-b (Supplementary Figures S4A, B). Interactions of cbl-b with key signaling molecules such as EGFR, AKT1, and MAPK1 were identified through the String database. To verify whether circ-0001454 regulates cbl-b, EGFR, AKT1, and MAPK1 by targeting miR-770-5p, we designed the following experiments based on existing research:Research has demonstrated that in an OVA-induced asthma mouse model, the use of EGFR or Src inhibitors can significantly improve peribronchial inflammation, airway remodeling, and airway hyperreactivity (El-Hashim et al., 2017). Similarly, Perilla seed decoction has been found to alleviate airway hyperreactivity in cough variant asthma model rats via the PI3K/AKT1/mTOR signaling pathway (Nguyen et al., 2023). Additionally, androgen receptors have been shown to inhibit the inflammatory response in allergic asthma airway epithelial cells through the modulation of MAPK1 and MAPK14 (Xia et al., 2022). To further explore whether circ-0001454 regulates cbl-b and influences EGFR, AKT1, and MAPK1 expression by targeting miR-770-5p, we executed additional functional rescue experiments. These experiments validated our hypothesis, confirming the regulatory impact of circ-0001454 through these signaling pathways. Meanwhile, in the asthma mouse model, cbl-b protein was highly expressed, while its downstream target genes showed low expression (Supplementary Figures S5A–G).

This research focused on miR-770-5p as a starting point and elucidated its role in asthma at the animal level. Through its interaction with circ-0001454, we further uncovered the regulatory function of circ-0001454 in asthma. By combining bioinformatics prediction and screening, we constructed the circ-0001454/miR-770-5p/cbl-b regulatory axis and validated the relationship among these molecules at both cellular and tissue levels, confirming that it aligns with the ceRNA mechanism, thereby regulating airway inflammation in asthma. Although this study has achieved significant findings, certain limitations remain. Due to constraints in experimental conditions and technical methods, only one cell line was used in this research. Future research could enhance the reliability of the results by incorporating multiple cell lines for validation. Additionally, the specific mechanism underlying the upregulation of circ-0001454 in HDM-induced BEAS-2B cells has not been fully explored. In subsequent studies, we plan to utilize 3D airway organoid models and conduct clinical experiments to further investigate the molecular mechanisms and provide more effective strategies for the prevention and treatment of asthma.

## Data Availability

The datasets presented in this study can be found in online repositories. The names of the repository/repositories and accession number(s) can be found in the article/Supplementary Material.
