# Supplementary material for: circ-0001454 alleviates asthma airway inflammation and remodeling via sponging miR-770-5p and regulating cbl-b
Source: Front Cell Dev Biol. 2025 Apr 8;13:1566223. doi: 10.3389/fcell.2025.1566223 (PMC12011828; doi:10.3389/fcell.2025.1566223)
Supplement: Supplementary file 1 [file DataSheet1.docx]

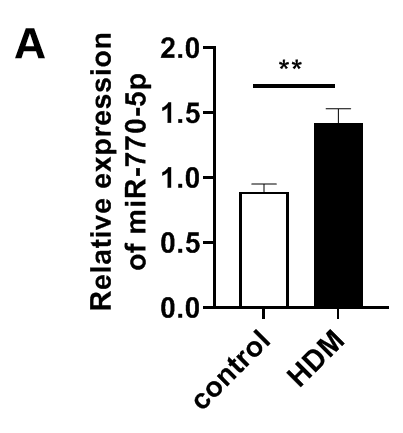


**FIGURE S1** Inhibition of miR-770-5p can alleviate airway inflammation in asthmatic mice**（A）** qPCR measures miR-770-5p in asthma animal model. Statistical significance denoted by ^**^*P* < 0.01 compared to control group.


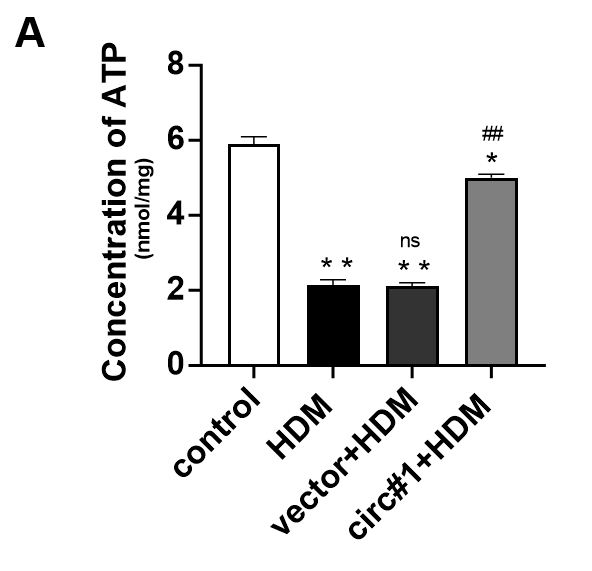


**FIGURE S2** circ-0001454 up-regulation reduce bronchial epithelial cell oxidative stress, apoptosis and mitochondrial membrane potential loss.**（A）**The expression of ATP in each group.

Statistical significance denoted by ^*^*P* < 0.05, ^**^*P* < 0.01 compared to control group; ^##^*P* < 0.01, n.s, compared to HDM group. n.s. for non-significant.


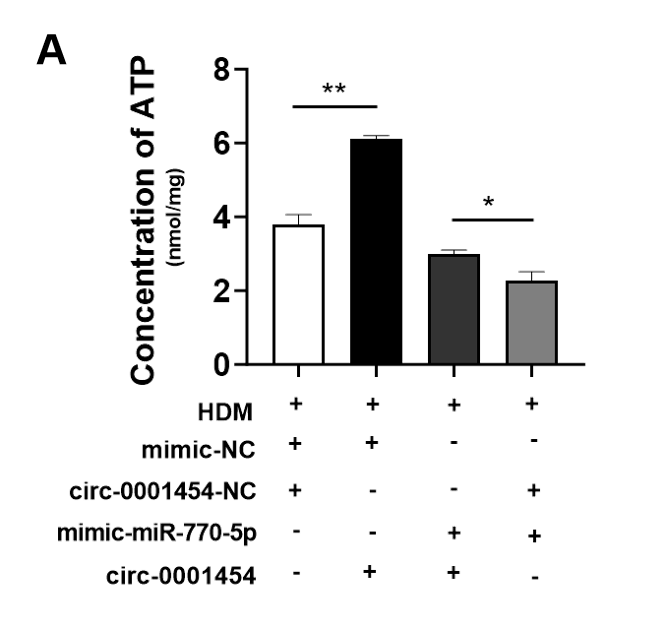


**FIGURE S3** circ-0001454 inhibits the expression of miR-770-5p, reducing oxidative stress, apoptosis, and the loss of mitochondrial membrane potential in bronchial epithelial cells**（A）**The expression of ATP in each group.Statistical significance denoted.by^*^*P* < 0.05, ^**^*P* < 0.01.


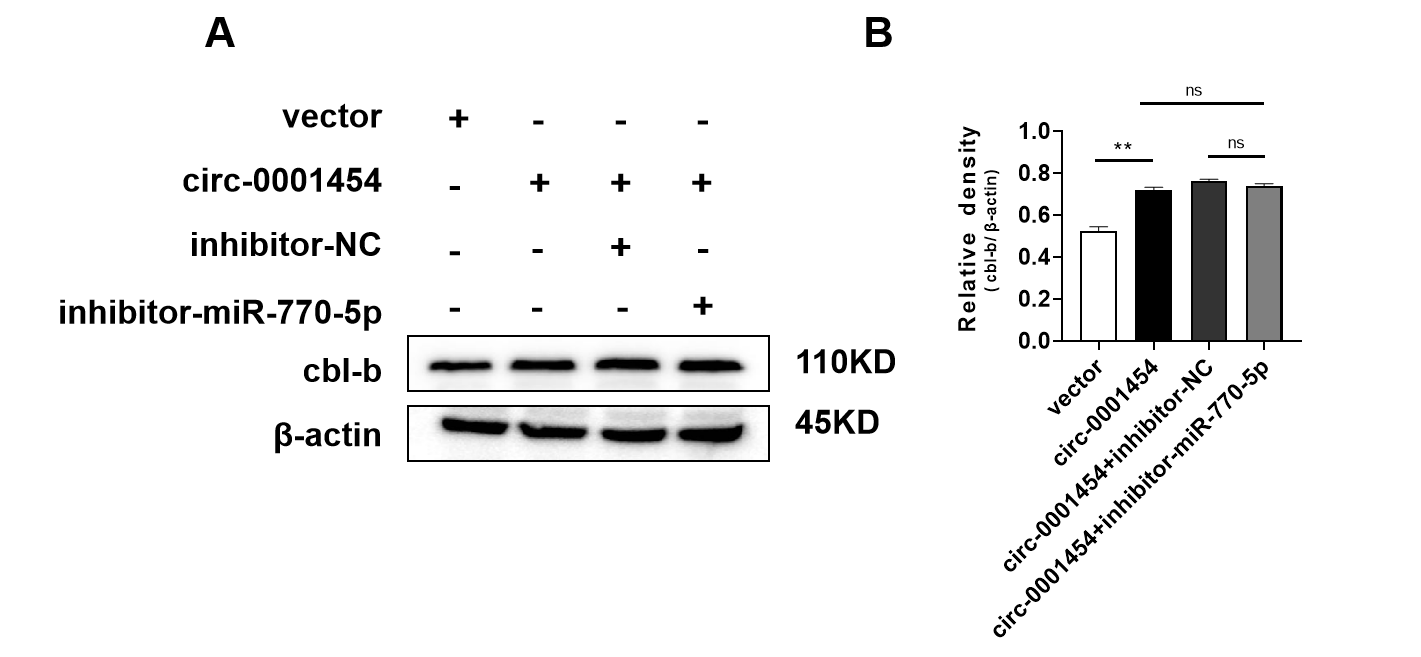
**FIGURE S4.** circ-0001454 targets miR-770-5p to upregulate cbl-b levels and alleviate airway inflammation.**（A-B）** Western blot analysis was conducted to investigate the direct regulatory effect of circ-0001454.Statistical significance denoted.by ^**^*P* < 0.01, n.s. for non-significant.


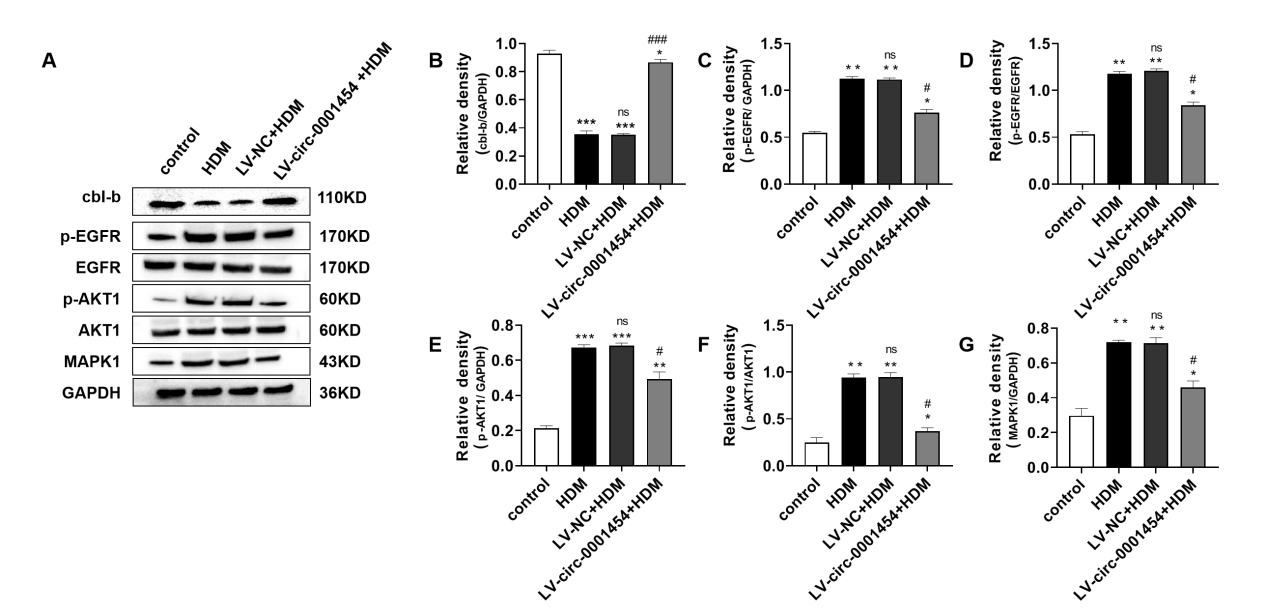


**FIGURE S5** Upregulation of circ-0001454 alleviates airway inflammation in asthmatic mice.**（A-G）** Western blot analysis was performed to detect the protein expression of cbl-b and controlled target genes in asthmatic mice.Statistical significance denoted by ^*^*P* < 0.05, ^**^*P* < 0.01, ^***^*P* < 0.001 compared to control group; ^#^*P* < 0.05, ^###^*P* < 0.001, n.s, compared to HDM group. n.s. for non-significant.
